# Poor drug adherence and lack of awareness of hypertension among hypertensive stroke patients in Kampala, Uganda: a cross sectional study

**DOI:** 10.1186/s13104-015-1830-4

**Published:** 2016-01-02

**Authors:** Isaac Mugwano, Mark Kaddumukasa, Levi Mugenyi, James Kayima, Edward Ddumba, Martha Sajatovic, Cathy Sila, Michael DeGeorgia, Elly Katabira

**Affiliations:** Mother Kevin Post Graduate Medical School, Nsambya Hospital, Uganda Martyr’s University, P. O. Box 5498, Kampala, Uganda; Department of Medicine, College of Health Sciences, Makerere University, P. O. Box 7072, Kampala, Uganda; Infectious Diseases Research Collaboration, Mulago Hill Road, MUJHU3 Building, P. O. Box 7475, Kampala, Uganda; Neurological and Behavioral Outcomes Center, University Hospital Case Medical Center, Case Western Reserve University, 11100 Euclid Ave, Cleveland, OH 44106 USA; I-Biostat, University of Hasselt, 3590 Diepenbeek, Belgium

**Keywords:** Stroke, Hypertension

## Abstract

**Background:**

Raised blood pressure (BP) remains an important risk factor for cardiovascular diseases such as stroke. Adherence to therapeutic recommendations especially antihypertensive drugs is important in BP control. The aim of the study was to assess the stroke risk factors and levels of adherence among hypertensive patients with stroke in Kampala Uganda.

**Methods:**

In a cross-sectional study we describe 112 hypertensive subjects with stroke from two Kampala city hospitals. A standardized pre-tested questionnaire was used to collect medical history, clinical details, radiological findings and laboratory data.

**Results:**

A total of 112 hypertensive subjects with stroke were enrolled between May 2013 and April 2014. The median ages were 63.5 years (52.5–75.0) for the cases. Seventy percent (78/112) of the study participants had ischemic strokes. Only 17 % were adherent to anti-hypertensive medications. The main cause of non-adherence appears to be lack of knowledge.

**Conclusions:**

Poor adherence of anti-hypertensive medications among hypertensive patients remains a big challenge in our setting. This has been attributed to lack of adequate knowledge and cost of the prescribed drugs. There is therefore an urgent need to promptly diagnose and educate hypertensive patients with emphasis on adherence to anti hypertensive drugs.

## Background

Stroke remains a global health problem with an estimated 15 million people worldwide suffering from non-fatal strokes yearly [1]. About 5.5 million people are estimated to have died from stroke in 2001 while a third of these developed disabilities [1–3]. Stroke is also the leading cause of functional impairment, with nearly 20 % of stroke survivors requiring institutional care after 3 months post stroke and 15–30 % remaining permanently disabled [4]. The incidence and mortality of stroke varies greatly among different populations and geographic locations. Stroke incidence has declined considerably in several western countries due to better preventive measures for hypertension. In contrast, stroke rates are increasing in sub-Saharan Africa, especially Uganda, where stroke awareness and therapeutic interventions are very limited [3]. The inter-stroke study reported important risk factors for stroke as history of hypertension, current smoking; waist-to-hip ratio; regular physical activity; diabetes mellitus; alcohol intake for more than 30 drinks per month or binge drinking; psychosocial stress; depression; cardiac causes; and a high ratio of apolipoproteins B to A1. These risk factors collectively accounted for 90.3 % (85.3–93.7) for all strokes [5]. Control of these major cardiovascular risk factors, using medical treatment and lifestyle changes has been advocated for so as to reduce the risk of stroke [6]. There is limited data regarding the stroke risk factors in sub-Saharan Africa. A major precipitant to stroke in sub-Saharan Africa is unrecognized and untreated hypertension. One study in Nigeria [7] looked at factors associated with stroke among hypertensive patients in Africa and found that 13.2 % of the stroke patients were first diagnosed as hypertensive at presentation with stroke.

Information on stroke risk factors among hypertensive patients will form a basis for designing specific stroke prevention strategies and treatment guidelines in our setting. This will be a key milestone in mitigating the rising incidence of stroke and stroke recurrences in Uganda. The overall objective of this study was to assess stroke risk factors and anti-hypertensive drug adherence among hypertensive patients admitted with stroke in two city hospitals in Kampala, Uganda.

## Methods

In a cross-sectional study design we consecutively recruited known hypertensive stroke subjects in two urban teaching hospitals in Kampala, Uganda.

The study was carried out in two hospitals, St Raphael of St Francis Nsambya and Mulago national teaching and referral hospital. Nsambya hospital is located 4.8 km south west of Kampala and is a tertiary hospital with a bed capacity of 361 beds and receives 8–10 stroke patients per month. Mulago is the National referral hospital for Uganda and also the teaching hospital for Makerere University College of Health Sciences. Mulago hospital has a 50 bed neurology unit. It receives over 30 stroke patients in a month. Both hospitals have resident neurologists with Computerized Tomography (CT) scan services. The study inclusion criteria were; adult hypertensive patients >18 years, with a radiological confirmed diagnosis of stroke on a CT scan and written informed consent. We excluded patients with Transient Ischemic attack (TIA), traumatic hemorrhagic stroke or patients with brain space occupying lesions such as tumors or metastases.

Over a period of 1 year (May 2013–April 2014), we consecutively enrolled hypertensive adults admitted with a diagnosis of an acute stroke and consented to participate in the study. Stroke was defined as a neurological deficit attributed to an acute focal injury of the central nervous system (CNS) by a vascular cause, including cerebral infarction, intracerebral hemorrhage (ICH) [8]. Ischemic stroke was defined as an episode of neurological dysfunction caused by focal cerebral, spinal, or retinal infarction while hemorrhagic stroke was defined as rapidly developing clinical signs of neurological dysfunction attributable to a focal collection of blood within the brain parenchyma or ventricular system that is not caused by trauma [8]. The strokes were confirmed by neurological exam demonstrating neurological deficits attributed to the involved area. On a non contrast CT a clearly visible hypo attenuated area represented ischemic stroke [9] while a hyper dense region when compared with the surrounding brain tissue showed a hemorrhagic stroke [10].

A general clinical examination, vital signs assessment including pulse, blood pressure (BP) and complete neurological examination were performed. The neurological exam included mental state exam, cranial nerve exam, muscle power, reflexes, tone and sensation. Gait was not performed among these participants. Peripheral blood was collected for fasting lipid profile, renal function tests, liver function tests, serum electrolytes and fasting blood glucose, complete blood count (CBC) and erythrocyte sedimentation rate (ESR). The level of blood sugar control was assessed by reviewing the medical record and determining the glycosylated haemoglobin levels at the time of recruitment into the study. Levels of glycosylated haemoglobin above 8 % were considered poor control while those below 7 % were considered good control. Patients were considered to have dyslipidemia if, they had total cholesterol ≥240 mg/dl, low-density lipoprotein cholesterol ≥160 mg/dl, <40 mg/dl) high-density lipoprotein cholesterol or self-reported prior diagnosis of high cholesterol and current use of cholesterol-lowering medications with supporting medical documents [11]. Appropriate referrals were done for study participants requiring specialized medical attention.

Using a standardized pre-tested questionnaire, demographic data and life styles history such as smoking, alcohol consumption and level of physical activity were obtained. We assessed for concurrent medical co-morbidities relevant to stroke such diabetes mellitus, Human Immunodeficiency deficiency (HIV), cardiac diseases and medication adherence. We also assessed smoking history (in pack years), level of moderate exercise based (walking, cycling, or gardening) and level of strenuous exercise (jogging, football, and vigorous swimming) for 4 h or more per week [7].

The Alcohol Use Disorders Identification Test (AUDIT) was used to categorize alcohol consumption among the study participants. The AUDIT categorizes alcohol use into excessive drinking, harmful drinking and alcohol dependence. It has a total of ten questions, three questions on alcohol consumption, three questions on drinking behaviors and dependence and four questions on the consequences or problems related to drinking. There are different scoring scales allocated to the questions. A score of less than four is categorized as low risk, 8–15 as risky or hazardous drinking, 16–19 as high risk or harmful and while those with scores of 20 or more are categorized as being alcohol dependent [12].

The Morisky 8-item Medication Adherence Questionnaire was used to assess the level of adherence to anti hypertensive medication by the participants. The questionnaire has a total of eight questions with a score of one allocated to each question. Participants are categorized as having high adherence if they score 0, 1 or 2 as medium adherence while those above two are categorized as having low adherence [13].

## Blood pressure

BP and heart rate were measured with an Omron automated sphygmomanometer model HEM-907 whose accuracy has been validated [14]. The admission systolic and diastolic BP were recorded among all study participants. Three readings were taken 3 min apart and the closest two were used to determine the averages which were recorded as the BP of the patient.

The standard of care provides for CT scan for all stroke patients, and then further work up for ECG and ECHO/carotid Doppler/CT angiography requires payments for these tests. All study subjects had an ECG done and those with irregular heart rates received further testing with Echocardiography.

## Data management and analysis

Data were entered into EPI-DATA version 2.1 (http://www.epidata.software.informer.com) and analysed using Stata 12.0 (Stata Corporation, College Station, Texas, USA). Summaries for frequencies, percentages, medians and interquartile ranges were presented in tables and figures.

## Ethical consideration and informed consent

Written informed consent was obtained from all study participants or next of kin/legal representative for individuals unable to complete written forms. Information about the study, its potential risks and benefits to the patients were elaborated to the patients/relatives in simple and concise language. Approval for conducting the study in the two hospitals was provided from the Department of Internal Medicine Mother Kevin Postgraduate Medical School and the School of Medicine, Research and Ethics Committee of Makerere University College of Health Sciences (Ref no—2013-147).

## Results

### Demographic characteristics of participants

A total of 336 stroke subjects were admitted during the study period with 62 and 268 from Nsambya and Mulago respectively. Out of these, 112 known hypertensive patients with stroke who provided written informed consent were then recruited during the study period, as illustrated in Table 1. The median age (IQR) was 63.5 (52.5–75.0) years. Nearly, 19 % (21/112) of the study subjects were less than 50 years. Nearly half of the study subjects were aged between 51 and 70 years. About 59 % (66/112) of the study participants were female. Eight percent of the study participants were single, 72 % were married, none cohabiting, 6 % divorced and 13 % widowed. Nearly half of the study participants lived within urban centers. The mean (±SD) systolic and diastolic admission BP in mmHg were 166 ± 33.2 and 98 ± 26.2 respectively.

**Table 1:** Demographic characteristics of study participants (n= 112)

Demographic characteristics	
Age in years, median (IQR)	63.5 (52.5–75.0)
Age categories	
≤40 years	7 (6.3)
41–50 years	14 (12.5)
51–60 years	26 (23.2)
61–70 years	26 (23.2)
71–80 years	24 (21.4)
>80 years	15 (13.4)
Gender, n (%)	
Male	46 (41.1)
Marital status, n (%)	
Single	9 (8.0)
Married	81 (72.3)
Cohabiting	0 (0.0)
Divorced	7 (6.3)
Widowed	15 (13.4)
Address, n (%)	
Rural	49 (43.7)
Urban	63 (56.3)
Occupation, n (%)	
Peasant farmer	51 (45.5)
Gainful employment	48 (42.9)
Retired or unemployed	13 (11.6)
Education level, n (%)	
None	32 (28.6)
1–7 years in school	52 (46.4)
8–13 years in school	6 (5.4)
>13 years in school	22 (19.6)

### Type and location of strokes among the study subjects

About 70 % (78/112) of the study subjects had ischemic stroke while 30 % (34/112) had a hemorrhagic stroke. Nearly, 92 % of the ischemic stroke occurred in the anterior cerebral circulation vessels compared to 8 % which occurred in the posterior circulation vessels.

### Hypertension care

The median years (IQR) for the duration of BP since hypertension diagnosis to the stroke incidents among the study subjects was 4.5 (2–10) years. Only 45 subjects (40.2 %) were regularly attending routine care for their hypertension control. More than half of the participants (64/112) had never sought medical attention nor had their BP reviewed again in the past 12 months after being diagnosed with high BP. Among those who regularly attended medical care for their hypertension, 29 % attended monthly, 5 % every two months and 9 % quarterly. Nearly 53 %, (59/112) were not currently on regular anti-hypertensive therapy. The most commonly prescribed therapies were calcium channel blockers (Amlodipine and Nifedipine) while the least prescribed was methyldopa at 0.9 %. More than half of the study subjects were receiving dual anti-hypertensive therapy, while 14 % were receiving three or more drugs for BP control. Only 2.6 % (3/112) of the study subjects were receiving lipid lowering therapy. However, 33 % (37/112) reported that they were taking concomitant herbal medication. Fifty-three percent had a family history of hypertension. Forty-two percent reported that the biggest challenge they faced was cost of the prescribed drugs, while 40 % had no challenges. Among the study participants 21 % (24/112) were diabetic, of whom over 95 % had a good level of blood sugar control (Table 2).

**Table 2:** Distribution of study participants by blood pressure variables (n = 112)

Blood pressure variable	n (%)
Duration since diagnosed with blood pressure (years), Median (IQR)	4.5 (2–10)
Routine care, n (%)	
Yes	45 (40.2)
Frequency of review, n (%)	
Not reviewed	64 (57.1)
Monthly	32 (28.6)
Every 2 months	6 (5.4)
Every 3 months	10 (8.9)
Current treatment for hypertension, n (%)	
Yes	53 (47.3)
Drugs participant uses for blood pressure, n (%)	
Calcium channel blockers	43 (39.1)
Diuretics	14 (12.7)
Β-blockers	15 (13.6)
ACEIs/ARBs	37 (33.6)
Alpha–adrenergic agonist (methyldopa)	1 (1)
Drug therapy, n (%)	
Mono-therapy	16 (28.1)
Duo-therapy	33 (57.9)
Multi-therapy	8 (14.0)
Lipid lowering therapy, n (%)	
Yes	3 (2.6)
Herbal medication, n (%)	
Yes	37 (33.0)
Family history of hypertension, n (%)	
Yes	59 (52.7)
Challenges experienced on drugs, n (%)	
Cost	47 (42.3)
Access	9 (8.1)
Side effects	4 (3.6)
Pill load	3 (2.7)
None	45 (40.5)
Previous diagnosis of diabetes mellitus, n (%)	
Yes	24 (21.4)
Currently on medication for diabetes, n (%)	
Yes	21 (87.5)
Level of control for sugar, n (%)	
Good	20 (95.2)
Poor	1 (4.8)

### Clinical characteristics, life-styles factors and anti hypertensive drug adherence

**Fig. 1:**
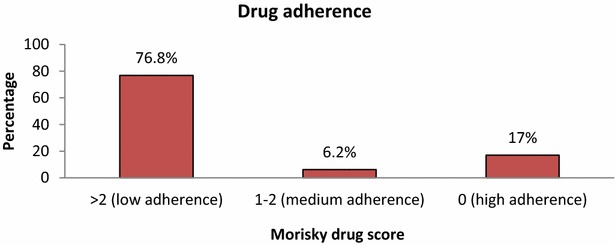
Shows the anti-hypertensive medication adherence according to the Morisky drug score

**Fig. 2:**
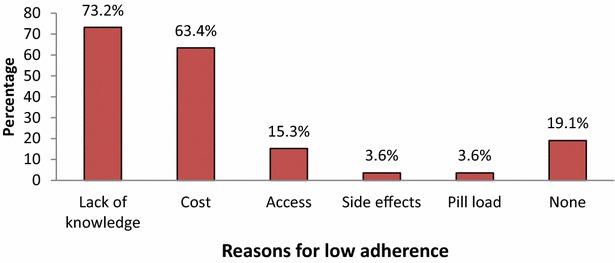
Shows the reasons for low drug adherence among the study participants

Eight study participants (7 %) had coexisting associated heart disease with atrial fibrillation contributing to 63 % (5/8) followed by ischemic heart disease (37 %). Nearly 4 % of the stroke cases (4/112) had coexisting HIV infection and three of these were already receiving anti-retroviral therapy (ART). Thirteen percent (14/112) had a previous stroke. Eight percent had a positive family history of stroke among their first degree relatives. Seventeen percent had a current history of alcohol consumption, more than half were considered low risk using the AUDIT score for alcohol. Only 32.1 % were involved in routine exercise (Tables 3, 4). Only 17 % of the study participants were highly adherent to their prescribed anti-hypertensive treatment compared to 77 % who were poorly adherent according to the Morisky drug scores (Fig. 1). The leading reasons for poor drug adherence were lack of knowledge of the chronicity of hypertension (73 %), cost of the drugs (63 %) and access to health care provision (15 %). However, 19 % of the study participants were not able to provide a reason for the poor drug adherence (Figs. 1, 2).

**Table 3:** History of clinical characteristics and life style among study participants (N = 112)

History of heart disease, n (%)	Yes	8 (7.1)
Previous diagnosis of HIV, n (%)	Yes	4 (3.6)
Previous history of stroke, n (%)	Yes	14 (12.5)
Family history of stroke, n (%)	Yes	9 (8.0)
Warfarin therapy, n (%)	Yes	3 (2.7)
Current history of smoking, n (%)	Yes	2 (1.8)
Current history of alcohol consumption, n (%)	Yes	19 (17.0)
Previous history of smoking, n (%)	Yes	3 (2.7)
Pack years, median (IQR)		10 (9–10)
Regular exercise, n (%)	Yes	36 (32.1)

**Table 4:** Distribution of study participants by their cholesterol levels (N = 112)

Total cholesterol, n (%)	
<200 (normal)	54 (60.0)
200–239 (border line high)	20 (22.2)
≥240 (high)	16 (17.8)
HDL cholesterol, n (%)	
>40 (desirable)	61 (67.8)
≤40 (lower than desired)	29 (32.2)
LDL cholesterol, n (%)	
<100 (optimal)	28 (31.1)
100–129 (near optimal)	24 (26.7)
130–159 (border line high)	23 (25.6)
160–189 (high)	9 (10.0)
≥190 (very high)	6 (6.7)
Fasting triglycerides, n (%)	
<150 (normal)	44 (80.0)
150–199 (border line high)	5 (9.1)
200–499 (high)	5 (9.1)
>500 (very high)	1 (1.8)

## Discussion

This cross-sectional descriptive study of hypertensive subjects with stroke underscores the magnitude of the lack of awareness of hypertension and the suboptimal medication adherence in urban Ugandans. Nearly two-thirds of the study participants were poorly adhering to the prescribed anti-hypertensive medications following the diagnosis of hypertension.

Hypertension still remains a major independent factor of morbidity and mortality [15]. With the raising levels of non-communicable diseases in sub-Saharan Africa the morbidity and mortality from cerebro-vascular accidents is estimated to increase [3]. Correct and prompt institution of BP reduction measures has shown benefit in reducing the incidence of stroke [16–18]. The lack of adhering to the prescribed medications for hypertension and stroke awareness within our communities might be increasing the high rates of strokes in Uganda. More than half of participants were not regularly attending medical care since being diagnosed with high BP. This would have predisposes the higher BP levels, inadequate control and lack of continuous health education subsequently increasing the risk of strokes. Up-scaling of hypertension awareness campaigns and health education is therefore urgently needed to stem this trend.

There are differences in age among people who suffer strokes within the sub-Saharan region compared to those in developed countries. In our study, 42 % of the study participants were less than 60 years. Studies have reported that stroke occurs at an earlier mean age of 57 years in sub-Saharan Africa compared to 66.0 years in developed countries, with those ≤45 years constituting 24 % in Africa and 8 % in developed countries [19, 20].

Antihypertensive medications are the mainstay of treatment for essential hypertension [21, 22]. Earlier studies have demonstrated that there is a significant relationship between lower medication adherence and first stroke among hypertensive patients [23–27]. In our study, 77 % of the study participants were poorly adhering to medication prior to stroke. Medication adherence is associated with improved BP control and with reductions in stroke among those at risk for stroke [24, 25]. Some of the study subjects were using concomitant herbal medications. Whereas some herbal medications have been reported to have hypotensive effects, some might interfere with hypertensive drug bioavailability and efficacy [28]. Recommending adherence to the correct drugs and dosage still remains a challenge in our settings.

Barriers to anti-hypertensive medication adherence are multi-factorial, including complex medication regimens, excessive dosing frequency or pill burden, personal behavioral factors, trust communications of the health personnel, drug side effects, complications of treatment and other co-existing medical conditions [29, 30]. Reasons reported by stroke survivors in other studies include; lack of adequate knowledge, cost of the medications, drug access, drug side effects and pill burden [31, 32]. The role of the health workers however, was not explored in this study. Health care workers need to ensure that patients understand their health condition, the importance of drug adherence and the implications of non-adherence. In Uganda, medications are typically provided for by the Ugandan government. However, during periods of drug stock-outs patients have to buy their medications out of pocket. Some hypertensive participants in our sample were poorly adhering due to financial or access reasons.

Overall, our findings suggest the need for increasing hypertension awareness and drug adherence in stroke prevention. Preliminary data from Rose et al. suggest that treatment intensification is ultimately the most effective strategy to achieve controlled BP regardless of the status of medication adherence [33, 34]. Setting up easily accessible BP measuring sites, and making treatment and support readily available, would help mitigate this upcoming scourge.

This study had important limitations including cross-sectional design and relatively small sample size. Medication adherence using patient self-reporting may introduce recall bias. Self-reported medication adherence may also be subject to social desirability bias and may lead to misclassification regarding the true prevalence of low medication adherence [35]. However adherence rates were similar to other studies and the Morisky scale is a well validated instrument that has been used for more than 25 years. Recent evidence demonstrates its correlation with pharmacy fill/refill data [36, 37]. We were also not able to assess the relationships between stroke and the co-morbidities such as diabetes mellitus, hyperlipidemia, HIV, other cardiovascular diseases, alcohol consumption, amphetamine use, cigarette smoking and level of physical activity due to the low power. No stroke severity scales were used in this study we hence did not measure the severity of the strokes in our participants. Another setback was absence of vascular imaging to classify the strokes based on the TOAST criteria, we relied on the radiologists reports which is has many setbacks. This made it difficult to accurately assess the location of strokes especially in the posterior fossa.

In conclusion, not being in routine care for hypertension and poor adherence to anti hypertensive medications are important factors among hypertensive patients admitted in the two study hospitals in Kampala, Uganda. There is an urgent need to raise the level of knowledge regarding screening for hypertension and the importance of BP control among hypertensive patients in Uganda. Increased funding to the Health Sector for the procurement of drugs and other health supplies could reduce the financial burden of out of pocket expenses met by hypertensive patients for routine care. There is also need for larger studies with a longitudinal design and more focus on lifestyle or other risk factors that may influence health behaviors and stroke risk.

## Abbreviations

AUDIT: the alcohol use disorders identification test
BP: blood pressure
CT scan: computerized tomography scan
MEPI: medical education partnership initiative
